# Inhibition of Protein Kinase CK2 Prevents Adipogenic Differentiation of Mesenchymal Stem Cells Like C3H/10T1/2 Cells

**DOI:** 10.3390/ph10010022

**Published:** 2017-02-09

**Authors:** Lisa Schwind, Sarah Schetting, Mathias Montenarh

**Affiliations:** Medical Biochemistry and Molecular Biology, Saarland University, Building 44, D-66424 Homburg, Germany; sasc23@gmx.de (S.S.); Mathias.Montenarh@uks.eu (M.M.)

**Keywords:** adipogenesis, transcription factors, protein kinase, kinase inhibitor

## Abstract

Protein kinase CK2 as a holoenzyme is composed of two catalytic α- or α’-subunits and two non-catalytic β-subunits. Knock-out experiments revealed that CK2α and CK2β are required for embryonic development. Little is known about the role of CK2 during differentiation of stem cells. Mesenchymal stem cells (MSCs) are multipotent cells which can be differentiated into adipocytes in vitro. Thus, MSCs and in particular C3H/10T1/2 cells are excellent tools to study a possible role of CK2 in adipogenesis. We found downregulation of the CK2 catalytic subunits as well as a decrease in CK2 kinase activity with progression of differentiation. Inhibition of CK2 using the potent inhibitor CX-4945 impeded differentiation of C3H/10T1/2 cells into adipocytes. The inhibited cells lacked the observed decrease in CK2 expression, but showed a constant expression of all three CK2 subunits. Furthermore, inhibition of CK2 resulted in decreased cell proliferation in the early differentiation phase. Analysis of the main signaling cascade revealed an elevated expression of C/EBPβ and C/EBPδ and reduced expression of the adipogenic master regulators C/EBPα and PPARγ2. Thus, CK2 seems to be implicated in the regulation of different steps early in the adipogenic differentiation of MSC.

## 1. Introduction

Mesenchymal stem cells (MSCs) are multipotent progenitor cells which can differentiate into adipocytes, chondrocytes, osteocytes, myocytes, and many other cell types [1]. Therefore, MSCs are promising therapeutic tools for tissue regeneration and repair. Understanding the molecular mechanisms underlying MSC differentiation seems to be important. Over the last few years, protein kinase CK2 turned out to play a key role in cell differentiation [2,3,4,5]. The serine/threonine kinase CK2 is implicated in a wide variety of different cellular processes including regulation of cell growth, cell proliferation, and apoptosis [6]. The cellular functions of CK2 are vital for proper cell function, organogenesis, embryogenesis, and organ homeostasis. In mammalian cells, CK2 is composed of two catalytic α- or α’-subunits and two non-catalytic β-subunits which form a tetrameric holoenzyme. In addition, there is increasing evidence for individual activities of the α- or β-subunits aside from the holoenzyme [7,8,9]. This notion is supported by the observation that various types of tissues express different levels of CK2α, CK2α’, or CK2β [9,10].

In mice CK2α knock-out is lethal by embryonic day 11 [3,11]. Knock-out mice for CK2β die at embryonic day 6 [12], whereas knock-out mice for CK2α’ are viable, but male mice are infertile due to a defective spermatozoa morphogenesis [13]. During mouse development, CK2 expression is high during organogenesis and considerably lower by birth [14]. Furthermore, earlier studies have shown a distinct role of CK2 during differentiation in several stem cell models for neuronal, osteogenic and hematopoietic differentiation [15,16,17,18,19,20]. Little is known about a role of CK2 in the differentiation of established mesenchymal stem cells to adipocytes [21].

The observation that the CK2 level and the CK2 kinase activity are elevated in cancer cells compared to normal, healthy cells has fuelled the search for specific inhibitors of the kinase activity in order to find tools for pharmacological treatment of cancer. There is a steadily increasing number of CK2 inhibitors published over the last 10 years, which can be classified as ATP analog, substrate analog, or compounds that inhibit the association of the CK2α and the CK2β subunit (for review see: [22,23]).

CX-4945 (5-(3-chlorophenyl)amino)benzo[c][2,6]naphthyridine-8-carboxylic acid), also known as Silmitasertib^®^, is a potent new-generation, orally available ATP-competitive inhibitor with a high specificity for CK2 [24,25]. CX-4945 has anti-proliferative activity in many cancer cell lines and is under clinical trials for cancer therapy [26,27].

Here, we have analyzed the effect of CX-4945 on the differentiation of the multipotent mesenchymal stem cell line C3H/10T1/2 into adipocytes. We conclude that CK2 positively affects adipogenesis in several ways early during differentiation.

## 2. Results

### 2.1. Characterization of CK2 and Adipogenic Transcription Factors during C3H/10T1/2 Differentiation

There is a limited number of models to study the differentiation of MSCs into adipocytes. One of the elegant model systems for this type of analysis is the MSC cell line C3H/10T1/2 [28]. To study adipogenesis in cell culture models like mouse embryo fibroblasts or the pre-adipocyte cell line 3T3-L1, a hormonal mixture—consisting of insulin, the glucocorticoid dexamethasone, and the phosphodiesterase inhibitor isobutyl-methyl-xanthine (IBMX)—is used. In order to set up the experimental conditions for the differentiation of C3H/10T1/2 cells, cells were treated with the same differentiation mix and monitored after a period of 12 days for the appearance of lipid droplets.

**Figure 1:**
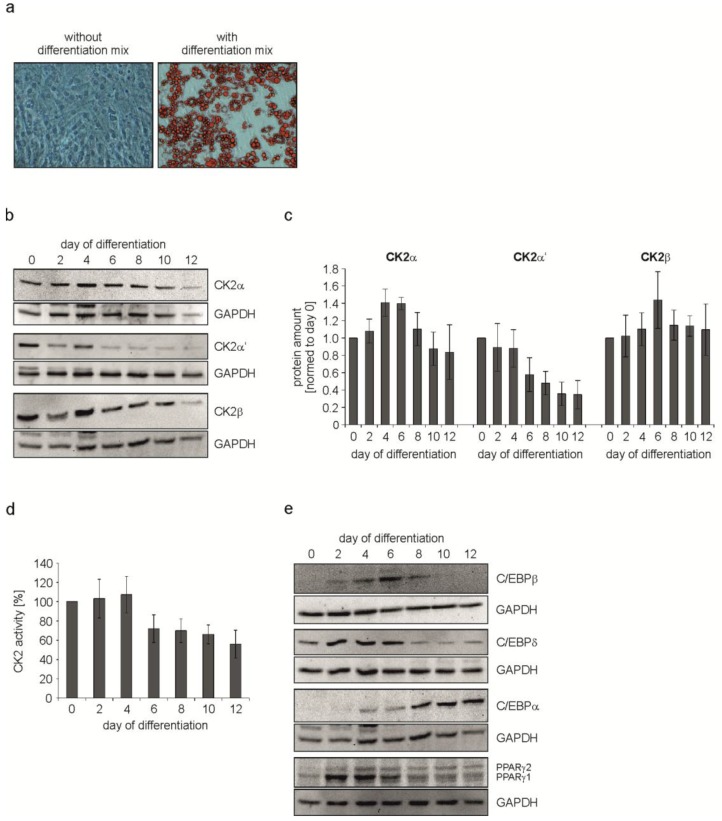
C3H/10T1/2 cells were differentiated for 12 days using IBMX, dexamethasone and insulin. (**a**) Bright field images showing the differentiation status at day 12 of differentiation. Control cells (without differentiation mix) were compared to cells differentiated with the mix. Lipid droplets were stained red with Oil Red O. Magnification 200×; (**b**,**e**) Cells were harvested at given time points during differentiation and protein expression was detected with specific antibodies using a Western Blot approach. GAPDH was used as loading control; (**c**) Quantification of the protein expression of CK2α, CK2α’, and CK2β normalized to GAPDH; (**d**) CK2 activity during adipogenic differentiation of C3H/10T1/2 cells over time shown by the incorporation of [^32^P]phosphate into the CK2 specific substrate peptide RRRDDDSDDD.

As shown in Figure 1a there were no signs of lipid droplets in untreated cells, whereas cells treated with the hormonal mix were full of droplets. These data show that the mixture of insulin, dexamethasone and IBMX is sufficient to induce differentiation of C3H/10T1/2 cells into adipocytes within 12 days. Next, we wondered whether there is an alteration in the expression of the CK2 subunits in the course of the differentiation process. Thus, differentiation was started as described above. Immediately before the start of differentiation (day 0) and then every two days, cells were lysed and the proteins in the cell extract were analyzed on a 12.5% SDS polyacrylamide gel followed by a transfer onto a PVDF membrane and Western blot with CK2α-, CK2α’-, and CK2β-specific antibodies. An antibody against GAPDH was used as a loading control. At day 4 there seems to be a slight increase in all three CK2 subunits, from day 6 on there was a continuous decrease in the level of CK2α and CK2β. CK2α’ was even faster downregulated after day 4 until the end of differentiation at day 12 (Figure 1b). The protein bands for CK2α, CK2α’, and CK2β were quantified from several experiments and the results are shown in Figure 1c. There was an increase for CK2α at days 4 and 6, whereas there was a continuous decrease in CK2α’. There was a slight increase in CK2β at day 6 followed by a decrease in the level of CK2β for the rest of the differentiation process. In order to analyze whether the decrease in the level of CK2 subunits might also correlate with a decrease in the protein kinase activity, we measured the CK2 kinase activity in the cell extract using the CK2 specific peptide substrate with the sequence RRRDDDSDDD [29]. As shown in Figure 1d, protein kinase activity at days 2 and 4 are mainly constant, whereas from day 6 onwards the kinase activity dropped to around 50% of the level in undifferentiated cells (day 0). Thus, in parallel to the decrease in the level of CK2α and CK2α’ proteins, the CK2 kinase activity also decreased. These results indicate that CK2 seems to be dispensable at late stages of adipogenesis.

It is well-known that adipocyte differentiation is largely controlled by two families of transcription factors, namely the CCAAT/enhancer binding proteins (C/EBPs) and the peroxisome proliferator-activated receptors (PPARs) [30]. Adipogenesis is characterized by a cascade expression of C/EBPα, C/EBPβ, C/EBPδ, PPARγ1, and PPARγ2. In order to analyze the influence of the CK2 level and its enzyme activity on the expression of these different transcription factors, we repeated the experiment described above but in this case cell extracts were analyzed for the expression of C/EBPs and PPARγ. GAPDH expression was analyzed as a loading control. C/EBPδ and C/EBPβ were transiently expressed between day 2 and day 8 of the differentiation process (Figure 1e). C/EBPα showed a constant increase in the expression in the course of the differentiation process. The expression of PPARγ2 increased from day 2 on, whereas PPARγ1 showed a maximal expression between day 2 and day 6.

From these results, we conclude that CK2 expression is high at the beginning of differentiation. Moreover, a programmed expression of different transcription factors is initiated from early time points of differentiation.

### 2.2. Inhibition of CK2 Kinase Activity with CX-4945 Influences Differentiation, Proliferation, and CK2 Protein Expression

From the experiments described above, it is tempting to speculate that CK2 might be necessary at the beginning of the differentiation process, but not in the second part of the differentiation. To prove this hypothesis, we attempted to study the influence of the inhibition of CK2 on the differentiation. We chose the inhibitor CX-4945 because it is highly specific for CK2, orally available and in clinical trials for the treatment of cancer [26,31,32]. The optimal concentration of the CK2 inhibitor has to be tested for every cell line. Therefore, we incubated proliferating C3H/10T1/2 cells either with DMSO alone (which is the solvent for CX-4945) or with 5, 10, 15, or 20 µM CX-4945. Kinase activity was tested with the synthetic substrate peptide [29].

**Figure 2:**
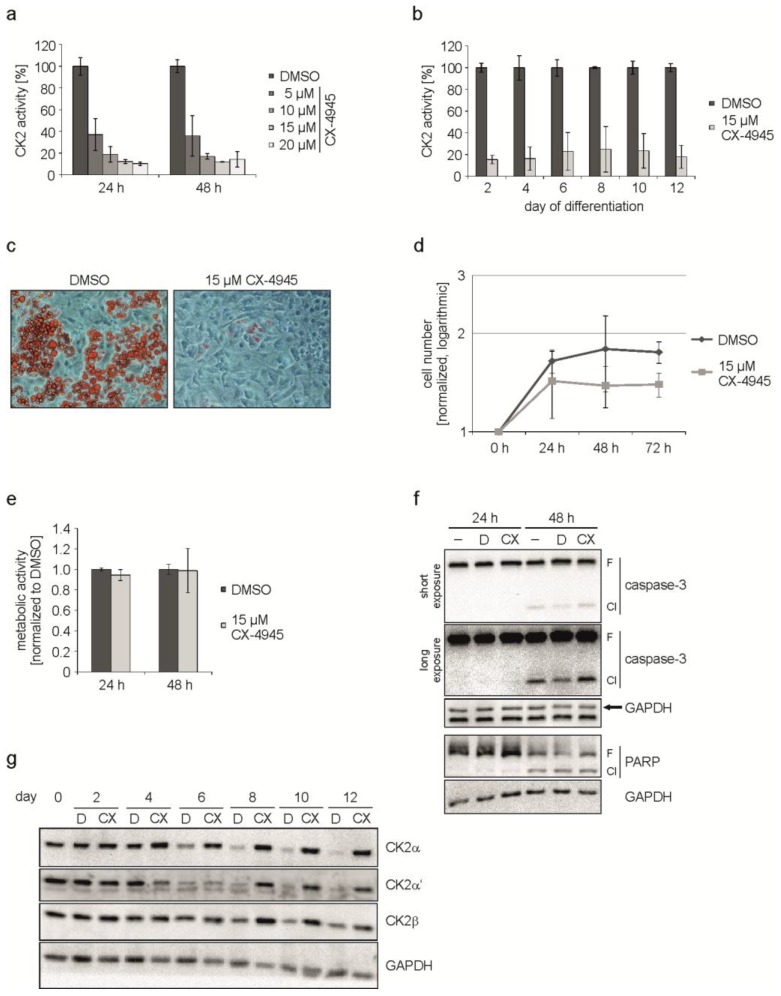
C3H/10T1/2 cells were differentiated in the presence of DMSO or CX-4945. (**a**) Proliferating C3H/10T1/2 cells were treated with 5, 10, 15, or 20 µM CX-4945 or DMSO as a control for 24 and 48 h. CK2 kinase activity was determined in an in vitro phosphorylation assay; (**b**) Cells were differentiated with differentiation mix containing DMSO or 15 µM CX-4945 and harvested at given time points. CK2 activity was determined in the protein extracts to confirm the inhibition over the complete differentiation process; (**c**) Differentiation status at day 12 of differentiation in cells treated with DMSO or 15 µM CX-4945. Lipid droplets were stained with Oil Red O and bright field images were recorded at 200× magnification; (**d**) Proliferation of DMSO- or 15 µM CX-4945-treated cells was determined at 0, 24, 48, and 72 h after the start of differentiation. Cell numbers were normalized to 0 h and are presented half-logarithmic; (**e**) Metabolic activity was examined in C3H/10T1/2 cells using MTT assay after differentiation of DMSO- or 15 µM CX-4945-treated cells for 24 or 48 h. Results were normalized to DMSO control values; (**f**) Differentiating cells were harvested 24 or 48 h after start of differentiation and cleavage of caspase-3 and PARP indicating apoptosis was examined using Western blot analysis −: untreated, D: DMSO-treated, CX: 15 µM CX-4945-treated, F: full length, Cl: cleavage product; (**g**) Western blot images of protein extracts from cells differentiated in the presence of DMSO (D) or 15 µM CX-4945 (CX) using specific antibodies for the CK2-subunits. GAPDH was used as loading control for all Western blots.

As shown in Figure 2a, there was a concentration-dependent reduction in the CK2 kinase activity ranging from 60% (5 µM CX-4945) up to 90% (20 µM CX-4945). For the following experiments we decided to use a concentration of 15 µM CX-4945, which resulted in a residual kinase activity of around 12%. To make sure, that CX-4945 was able to reduce the CK2 kinase activity during the whole differentiation process, we determined the activity from day 2 to day 12 of the differentiation. We found that the kinase activity was reduced by 75% to 85% in the presence of CX-4945 compared to cells without inhibitor over the whole period of differentiation (Figure 2b). In the next step, cells were incubated with the differentiation mix either in the presence or absence of 15 µM CX-4945 and the appearance of lipid droplets was monitored on day 12 of differentiation. As shown in Figure 2c, cells treated with the hormonal mix exhibited multiple lipid droplets, whereas in the presence of CX-4945 there were virtually no droplets. Thus, inhibition of CK2 by CX-4945 inhibits differentiation of the MSC cell line C3H/10T1/2 into adipocytes. Furthermore, cells looked healthy after treatment with CX-4945 and no morphological changes associated with apoptosis could be observed. This observation is in contrast to the effect of CX-4945 in many cancer cells [26,32].

It is known that 3T3-L1 pre-adipocytes respond to the treatment with the differentiation mix with mitotic clonal expansion (MCE) and that it is crucial for differentiation [33]. On the contrary, human MSC do not need MCE [34] and nothing is known about it in C3H/10T1/2 cells. Therefore, we asked whether CK2 inhibition might have an influence on the proliferation of C3H/10T1/2 cells. Cells were grown in the absence or presence of CX-4945 and counted at 0, 24, 48, and 72 h after the start of differentiation. The growth curve analysis, which is shown in Figure 2d, revealed a growth reduction in the presence of CX-4945. Thus, there seems to be an effect of CK2 inhibition on cell growth before the entrance into differentiation. A reduction in proliferation due to CX-4945 treatment could be the result of disturbances in cellular metabolism or apoptosis induction. So subsequently, we used an MTT assay to analyze metabolic activity 24 and 48 h after start of differentiation. As presented in Figure 2e, the metabolism of the cells was only marginally influenced by CX-4945-treatment compared to DMSO control. Apoptosis induction was examined by the detection of caspase-3 and one of its substrates poly [ADP-ribose] polymerase (PARP), which are cleaved in the event of apoptosis. Figure 2f shows that a part of caspase-3 and PARP full length proteins were cleaved 48 h after the start of differentiation, independently of the kind of treatment. Treating the cells with CX-4945 did not significantly enhance cleavage. From this experiment we conclude that the observed reduction of proliferation is not caused by metabolic disturbance or apoptosis, but by CK2 inhibition.

Since we have shown above that CK2 inhibition by CX-4945 resulted in impaired adipogenic differentiation, we wondered whether this had an influence on CK2 subunit expression. C3H/10T1/2 cells were treated with the hormonal mix to induce differentiation in the presence or absence of the CK2 inhibitor CX-4945. Proteins were extracted at different time points up to day 12 of differentiation and analyzed for CK2α, CK2α’, and CK2β subunits. The Western blot shown in Figure 2g shows that there is a reduction in CK2α, CK2α’, and CK2β expression during the differentiation, which is in agreement with the results shown in Figure 1b, whereas in the presence of the CK2 inhibitor (CX) no reduction could be observed. These results show that downregulation of CK2 subunits is accompanied with adipogenic differentiation. Inhibition of the kinase activity of CK2 prevents differentiation. In this case, the levels of the CK2 subunits remain constantly high.

### 2.3. Inhibition of CK2 Activity with CX-4945 Influences Expression of Important Adipogenic Transcription Factors

In Figure 1e, we have shown that differentiation of C3H/10T1/2 cells is accompanied by a specific expression of transcription factors. Therefore, in the next experiment we asked whether inhibition of CK2 with CX-4945 might influence the expression of these transcription factors. Thus, C3H/10T1/2 cells were incubated with the differentiation mix either in the presence or absence of CX-4945. Cells were harvested at different time points after the start of differentiation and proteins in the cell extracts were analyzed by SDS-polyacrylamide gel electrophoresis followed by Western blot with specific antibodies against C/EBPα, C/EBPβ, C/EBPδ, and PPARγ2. In this experiment, we focused on the PPARγ2 isoform, because this is the one responsible for adipogenesis [35,36].

**Figure 3:**
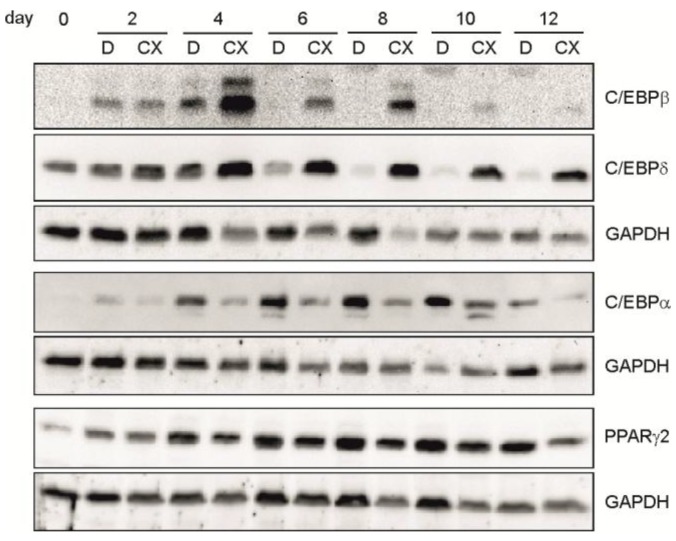
C3H/10T1/2 cells were differentiated in the presence of DMSO (D) or 15 µM CX-4945 (CX) and harvested at given time points during differentiation. Proteins were extracted and separated on a 12.5% SDS-polyacrylamide gel and transferred to a PVDF-membrane. C/EBPβ, C/EBPδ, C/EBPα, and PPARγ2 were detected with specific antibodies. GAPDH was used as a loading control.

As shown in Figure 3, in the presence of CX-4945, we observed an elevated and prolonged expression of C/EBPβ and C/EBPδ as well as a reduced expression of C/EBPα and PPARγ2 compared to the regular differentiation (DMSO). Thus, in addition to the influence on cell growth at early stages of differentiation, these results point to an influence of the CK2 kinase activity on the expression of the early transcription factors C/EBPβ and C/EBPδ, which then influence C/EBPα and PPARγ2 expression, leading finally to a block in differentiation.

## 3. Discussion

Obesity is a common health problem in industrialized countries, which is closely associated with other diseases such as hypertension, cancer, diabetes, and atherosclerosis just to mention a few. Therefore, there is an acute need for new and effective strategies to reduce obesity. Here, we have used the mouse embryo mesenchymal stem cell line C3H/10T1/2 which was first described in 1973 [28]. These cells behave in a manner similar to that of mesenchymal stem cells, making C3H/10T1/2 cells a useful model for cell differentiation. C3H/10T1/2 cells were stimulated either with BMP2 or with peptides derived from different regions of the bone morphogenetic protein receptor type Ia (BMPRIa) and then evaluated for their chondrogenic and osteogenic potential. The peptides were identified as inhibitors of the binding of CK2 to BMPRIa [16]. These peptides activate the BMP signaling in the absence of BMP [37]. Here, we have used a mix of dexamethasone, IBMX, and insulin to induce adipogenic differentiation. This process can be monitored by the appearance of lipid droplets which is more or less completed at day 12 after the start of differentiation. The differentiation process is governed by the regulated expression of several transcription factors such as C/EBPα, C/EBPβ, C/EBPδ, and PPARγ. In addition to the regulated expression of these transcription factors we also observed changing levels of the CK2 subunits. Interestingly, the catalytic CK2α’ subunit was downregulated rapidly during differentiation whereas CK2α and CK2β initially increased and then slowly decreased. These results indicate that at least CK2α’ is not necessary for differentiation. The reduction of the level of the CK2 subunits is accompanied by a decrease in the protein kinase activity. Therefore, we conclude that neither the CK2 subunits nor the enzymatic activity of CK2 is required towards the end of differentiation. The CK2 protein levels as well as the CK2 kinase activity are high at the beginning of differentiation. By immunohistochemistry and by in situ hybridization CK2 protein levels as well as mRNA levels for the individual subunits were found to be elevated in mouse embryogenesis [38]. Similar observations were made for rat, chicken, and *C. elegans* embryonic development [39,40,41]. An enhanced expression of CK2 goes along with an elevated CK2 kinase activity with a maximum at day 11 [14]. Knock-out experiments revealed that CK2α^−/−^ as well as CK2β^−/−^ mice are embryonically lethal [3,12]. The role of CK2 during differentiation of stem cells is less clear [17,18,19]. It was recently shown that in the pre-adipocyte cell line 3T3-L1 CK2 is necessary for early steps in differentiation which is in agreement with our present observation with C3H/10T1/2 cells [42]. An early step in differentiation of 3T3-L1 cells is one or two rounds of cell division prior to differentiation and it may well be that CK2 is necessary for this early step. Our present analysis of the cell growth properties showed that there is indeed an influence of CX-4945 on cell growth. This observation is compatible with the role of CK2 in proliferation. It is well known that the CK2 level and CK2 activity is high in rapidly proliferating cells such as tumour cells and low in healthy normal cells [43,44]. Over the last 10 years this particular observation has fuelled the search for specific inhibitors of the CK2 kinase activity in order to find a new therapeutic approach for the treatment of cancer. Among the numerous inhibitors now published for CK2 we have chosen CX-4945 because this inhibitor is bioavailable and used in clinical trials for the treatment of cancer [24,32]. As shown here, CX-4945 inhibits the differentiation of C3H/10T1/2 cells into adipocytes. This inhibition goes along with an early induction of the level of C/EBPδ and C/EBPβ. C/EBPδ interacts with C/EBPβ to induce C/EBPα and PPARγ2 expression during adipogenesis [45]. Our data, however, show that an elevated level of C/EBPβ and C/EBPδ is not sufficient to stimulate the expression of C/EBPα and PPARγ2, which are the master regulators of adipogenesis [35,46]. In contrast, there is a reduction in the level of C/EBPα and PPARγ2 in the presence of the CK2 inhibitor. One reason for this observation may be a missing CK2 phosphorylation of C/EBPδ and/or C/EBPβ. It was indeed recently shown that C/EBPδ is phosphorylated by CK2 [47]. Although the binding of C/EBPδ to C/EBPβ is not influenced by the CK2 phosphorylation of C/EBPδ, it was shown that the CK2 phosphorylated C/EBPδ transactivated the PPARγ2 promoter better than the non-phosphorylatable C/EBPδ mutant. This observation is in a good agreement with the results shown here and may explain them.

**Figure 4:**
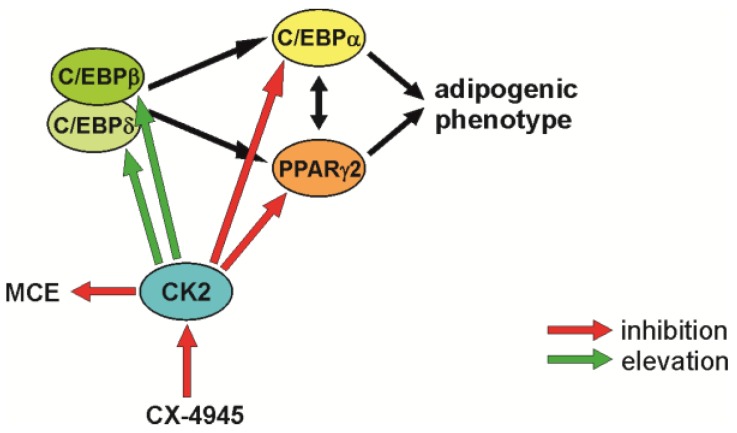
Schematic outline of the observations on adipogenic differentiation after inhibition of CK2 with CX-4945.

In summary, we would like to propose the model shown in Figure 4. CK2 seems to have a triple influence on early steps during the differentiation of mesenchymal stem cells. First, inhibition of the CK2 kinase activity reduced cell proliferation; second, CK2 inhibition increased the level of the two transcription factors C/EBPβ and C/EBPδ; and third, CK2 inhibition leads to a reduction in the expression of PPARγ2 and C/EBPα, two transcription factors, which are absolutely necessary for the differentiation into adipocytes.

## 4. Materials and Methods

### 4.1. Cell Culture, Differentiation, and Treatment of Cells

The C3H/10T1/2 cell line (ATCC: CCL-226™) was isolated from a line of C3H mouse embryo cells [28]. This cell line was kindly provided by Angelika Barnekow, Münster. C3H/10T1/2 cells can be induced to terminally differentiate into adipocytes by the addition of different hormones or chemical agents. Cells are maintained in Dulbecco’s modified Eagle’s medium (DMEM; Thermo Fisher Scientific, St. Leon-Rot, Germany) supplemented with 10% (*v*/*v*) fetal calf serum (FCS) in a humidified atmosphere containing 5% CO_2_ at 37 °C.

To differentiate C3H/10T1/2 cells into adipocytes, cells were seeded at a density of 2 × 10^4^ cells/cm^2^. When cells were two days post-confluent, medium was removed and substituted by the differentiation mix I (DMEM + 10% FCS, 0.5 mM IBMX, 0.25 µM dexamethasone, 5 µg/mL insulin) (corresponds to day 1 of the differentiation). After three days, mix I was replaced by fresh medium. From day six on, cells were incubated with differentiation mix II (DMEM + 10% FCS, 5 µg/mL insulin), which was replaced every three days.

The CK2 inhibitor CX-4945 (Selleckchemicals, Munich, Germany) was dissolved in dimethyl sulfoxide (DMSO; Merck, Darmstadt, Germany) to a 10 mM stock solution, which was used to treat the cells in a final concentration of 15 µM throughout the entire differentiation unless otherwise stated. In control experiments we used the same volume of the solvent DMSO alone.

### 4.2. Determination of Proliferation and Metabolic Activity

Cells were seeded in 24-well plates (5 × 10^4^ cells/well). After three days, cells were differentiated in the presence of DMSO or 15 µM CX-4945. To determine proliferation, cells were counted in triplicates using a Neubauer chamber (factor: 10^4^) at 0, 24, 48 and 72 h after start of differentiation. Cells were trypsinized and resuspended in a small amount of cell culture medium. A small amount of the cell suspension was mixed with an equal amount of the diazo dye trypan blue to stain dead cells. To determine the proliferation rate, only living cells were counted. The cell number determined for 0 h was set to 1.

To determine metabolic activity, cells were incubated for 1 h with 3-[4,5-dimethylthiazol-2-yl] 2,5-diphenyl tetrazolium bromide (MTT) reagent (1 mg/mL solved in PBS) at 24 and 48 h after start of differentiation. Subsequently, cells were lysed with solubilization solution (10% SDS, 0.6% acetic acid in DMSO) and absorption was measured at 595 nm. Results were normalized to DMSO.

### 4.3. Extraction of Proteins

Cells were washed with cold PBS, scraped off the plate, centrifuged (250× *g*, 4 °C) and the pellet was extracted with RIPA-buffer (50 mM Tris-HCl, 160 mM NaCl, 0.5% sodium desoxycholate, 1% Triton X-100, 0.1% SDS, pH 8.0) in the presence of protease inhibitors (Complete™, Roche Diagnostics, Mannheim, Germany). After 30 min on ice, cells were sonicated (3 × 30 s). After lysing, the cell debris was removed by centrifugation at 13,000× *g*. The protein content was determined with the BioRad reagent dye (BioRad, Munich, Germany). Protein extracts were immediately used for Western Blot analysis or for in vitro phosphorylation.

### 4.4. SDS–Polyacrylamide Gel Electrophoresis and Western Blot Analysis

Proteins were analyzed by SDS-polyacrylamide gel electrophoresis according to the procedure of Laemmli [48]. Proteins dissolved in sample buffer (130 mM Tris-HCl, pH 6.8, 0.02% bromophenol blue, 10% β-mercaptoethanol, 20% glycerol, 4% SDS) were separated on a 12.5% SDS- polyacrylamide gel in electrophoresis buffer (25 mM Tris-HCl, pH 8.8, 192 mM glycine, 0.1% SDS) and transferred onto a PVDF membrane (Roche Diagnostics, Mannheim, Germany) in a buffer containing 20 mM Tris-HCl, 150 mM glycine, pH 8.3. The membrane was blocked with 5% dry milk in TBS-Tween 20 for one hour and then incubated with appropriate primary antibodies diluted in TBS-Tween 20 with 1% dry milk or 5% bovine serum albumin (BSA) according to the supplier’s instructions. The membrane was washed twice with the incubation buffer and incubated with the peroxidase-coupled secondary antibody (anti-rabbit 1:30,000 or anti-mouse 1:10,000 in incubation buffer) for 1 h. Signals were visualized by the Lumilight system of Roche Diagnostics (Mannheim, Germany).

For the detection of protein kinase CK2 we used rabbit anti-peptide sera #26 (α-subunit), #30 (α’-subunit) and #32 (β-subunit) [49] and the mouse monoclonal antibody 6D5 (β-subunit) [50] or antibody 1A5 (α-subunit) [51]. C/EBPβ- (#3087), PPARγ- (#2430), caspase-3- (#9662), and PARP- (#9542) specific antibodies were from Cell Signalling Technology, the C/EBPδ-specific antibody (sc-151) was purchased from Santa Cruz Biotechnology Inc. (Heidelberg, Germany) and the C/EBPα- (ab40764) and PPARγ2- (ab45036) specific antibodies from Abcam (Cambridge, UK). Furthermore, we used a GAPDH specific antibody (sc-25778) from Santa Cruz Biotechnologies as loading control.

### 4.5. CK2 In Vitro Phosphorylation Assay

To determine the kinase activity of CK2, cells were lysed and the extracts were used in a kinase filter assay. In this assay, we measured the incorporation rate of [^32^P]phosphate into the synthetic CK2 specific substrate peptide with the sequence RRRDDDSDDD [29]. Twenty µL kinase buffer (50 mM Tris-HCl, pH 7.5, 100 mM NaCl, 10 mM MgCl_2_, 1 mM DTT) containing 30 µg proteins were mixed with 30 µL CK2 mix (25 mM Tris-HCl, pH 8.5, 150 mM NaCl, 5 mM MgCl_2_, 1 mM dithiothreitol (DTT), 50 µM ATP, 0.19 mM substrate peptide) containing 10 µCi/500 µL [^32^P]-γ-ATP. The mixture was spotted onto a P81 ion exchange paper. The paper was washed with 85 mM H_3_PO_4_ three times. After treatment with ethanol the paper was dried and the Čerenkov-radiation was determined in a scintillation counter.

### 4.6. Staining of Lipid Droplets in C3H/10T1/2 Cells

For staining lipids with Oil Red O (Sigma Aldrich, Munich, Germany), cells were grown in six-well plates and left untreated or differentiated into adipocytes as described above. After removing the medium and washing with PBS, cells were fixed in 3.7% formaldehyde in PBS for 2 min at room temperature and then washed three times with PBS. Oil Red O was dissolved in isopropanol (0.5 g/100·mL). A 60% solution of Oil Red O was prepared with bi-distilled water, incubated for 10 min at room temperature, and subsequently filtered through a folded filter (3MM, Whatman, UK). Cells were incubated with Oil Red O (2 mL/well) for one hour at room temperature. After incubation, the Oil Red O was removed and cells were washed twice with distilled water. The staining of the lipid droplets was analyzed by phase contrast microscopy.
